# Case of Osteo-Sarcoma of the Humerus, Necessitating Amputation at the Shoulder Joint

**Published:** 1862-03

**Authors:** C. Winne

**Affiliations:** Buffalo


ART. III.—*Case of Osteo-Sarcoma of the Humerus, necessitating amputation at the shoulder joint, by* C. Winne, M. D., *Buffalo.*

I submit for admission into your columns the following history of malignant disease of the humerus and surrounding structures, that necessitated its amputation at the shoulder joint:

A German laborer at forty, had for some years prior to the period when seen by me, suffered from deep and severe lancinating pain in the arm about its middle and near the insertion of the deltoid muscle. From his account of the case we learn that there first appeared a small, hard tumor in the above mentioned spot; this progressively increased and became more painful, changing from a color similar to that of the neighboring integuments to a deep red, permeated by veins which were often turged with blood, and from a uniform spherical shape, smooth on its surface, to a lobulated ovoid form, and when examined by the touch having alternations of hard and elastic and compressible spaces, that denoted an organization not altogether homogeneous, and resembling those that are classed under the designation of osteo-sarcomatous tumors. It conformed very closely to the description given by Dr. Warren in his treatise on this subject, having the three constituents.; 1st, the bone on which the swelling was developed; the periosteum and the soft medulary portion contained in its structure

From inspection and manipulation it seemed to have emerged from the cancelated structure, thrusting before in the exterior and periosteum, and as the latter was extended before its increasing growth, small plates like fish scales of an irregular shape and various sizes were deposited in its surface, until the tumor attained the magnitude of a very large orange measuring perhaps eight inches by six in circumference. The health of this man had deteriorated very rapidly in a few months before he was operated upon; the surface of the tumor had ulcerated in several places, and frequent hemorrhages had ensued. At length sloughing of the protruding mass had took place, and a large portion of the tumor separated, leaving an immense fœted, ulcerated and bleeding surface, that threatened to destroy him by exhaustion, if relief was not obtained.

The malignant character of the disease was beyond a doubt, and as the axiliary glands were but little implicated, and as in the course of the operative proceedure it was hoped that they could be removed; the operation was soon commenced and completed without accident or loss of blood. The structure of the dislocated mass presented every indication of a malignant nature; it had evidently originated in the spongy tissue of the humerus, and in its out-growth had pushed every structure before it, causing various concretions to be deposited by the periosteum, and changing the natural condition of the muscles and cellular tissue partly to a fibro-cartilaginous and partly medullary condition. At that period, 1847, we had not the means of making a microscopic investigation, and could only judge from its color, density, history, &c. The process of cicatrizing went on as in any ordinary amputation, and we supposed that the danger of any recurrence of malignant disease attacking the wound was over, but about the third week at the angle where two flaps joined there appeared at first some florid granulations that did not yield to pressure nor caustics; the fungoid mass increased rapidly, attained the size of a pigeon's egg, and became changed in color to a deep purple. It required the application twice of the arsenical paste, before this fungus was reduced to a level with the skin; it at last cicatrized soundly, and gave no further alarm about its return. The patient gradually recovered tolerable health, and survived the operation about fifteen months, when, as we learned some length of time after his death, he fell a victim to congestive pneumonia.

